# Targeting M2-like tumor-associated macrophages is a potential therapeutic approach to overcome antitumor drug resistance

**DOI:** 10.1038/s41698-024-00522-z

**Published:** 2024-02-10

**Authors:** Shujing Wang, Jingrui Wang, Zhiqiang Chen, Jiamin Luo, Wei Guo, Lingling Sun, Lizhu Lin

**Affiliations:** 1 The First Clinical Medical School of Guangzhou University of Chinese Medicine, Guangzhou, China; 2 The First Affiliated Hospital of Guangzhou University of Chinese Medicine, Guangzhou, China; 3 Guangdong Clinical Research Academy of Chinese Medicine, Guangzhou, China; 4 Lingnan Medical Research Center, Guangzhou University of Chinese Medicine, Guangzhou, China

**Keywords:** Cancer microenvironment, Oncology, Tumour immunology

## Abstract

Tumor drug resistance emerges from the interaction of two critical factors: tumor cellular heterogeneity and the immunosuppressive nature of the tumor microenvironment (TME). Tumor-associated macrophages (TAMs) constitute essential components of the TME. M2-like TAMs are essential in facilitating tumor metastasis as well as augmenting the drug resistance of tumors. This review encapsulates the mechanisms that M2-like TAMs use to promote tumor drug resistance. We also describe the emerging therapeutic strategies that are currently targeting M2-like TAMs in combination with other antitumor drugs, with some still undergoing clinical trial evaluation. Furthermore, we summarize and analyze various existing approaches for developing novel drugs that target M2-like TAMs to overcome tumor resistance, highlighting how targeting M2-like TAMs can effectively stop tumor growth, metastasis, and overcome tumor drug resistance.

## Introduction

The 2020 data revealed approximately 19.3 million new instances of tumors and around 10 million deaths linked to tumors worldwide. Both incidence and death rates have been quickly rising globally^1^. Drug therapy is still a primary clinical treatment for malignant tumors. However, the issues of the poor treatment outcomes, tumor progression, and poor prognosis caused by drug resistance have always been difficult to solve. Consequently, the study of tumor drug resistance has risen to prominence in the clinical management of tumors today. Contemporary research found that tumor drug resistance stems from the interaction of two critical factors, the intratumor heterogeneity and the immunosuppressive nature of the TME^2^. TME is composed of diverse cellular elements, including lymphocytes, extracellular matrix, growth factors, cytokines, chemokines, lymphocytes, TAMs, dendritic cells, natural killer cells, and myeloid-derived suppressor cells. The essential elements of TME are TAMs, which comprise two mutually polarizable subtypes: M1-like TAMs and M2-like TAMs. M2-like TAMs are essential for malignant metastasis, invasion and treatment resistance^3,4^. Their significant role emphasizes their potential as targets to overcoming tumor drug resistance. This review provides a synthesis of the mechanisms for modulating M2-like macrophages to surmount resistance to antitumor therapy, along with an overview of clinical trials based on these related mechanisms. Also, we analyze the future development potential of this novel therapeutic strategy.

## Biological feature of TAMs

### The origin of TAMs

**Fig. 1:**
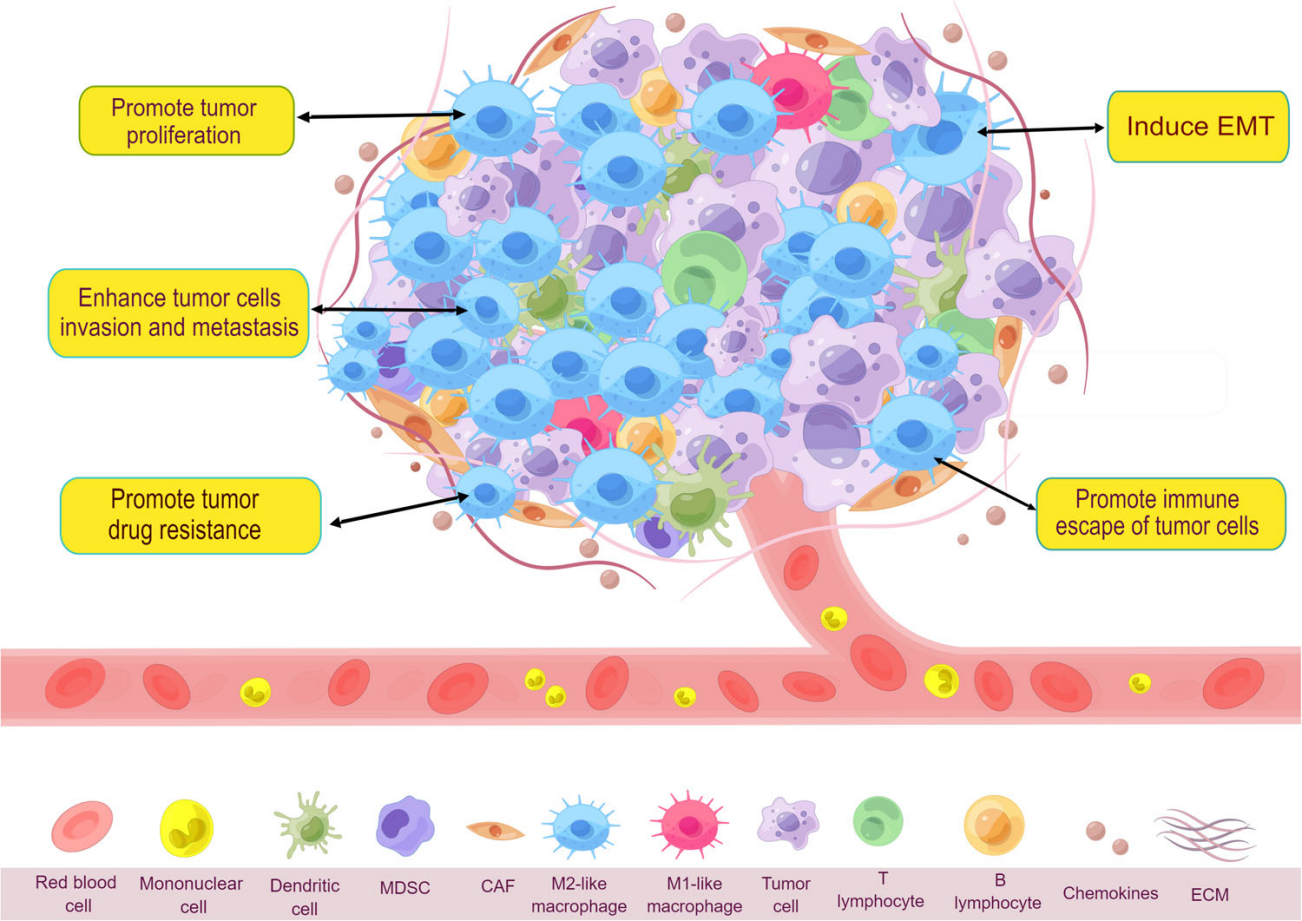
TAMs are the most critical components of the TME. TAMs promote many malignant progressions of tumors by regulating the TME, such as tumor proliferation, immune evasion by tumor cells, EMT, tumor invasion, tumor metastasis, tumor drug resistance. (By Figdraw).

As tumor cells interact with the extracellular interstitium during their growth, they create a particular environment conducive to their proliferation. This environment is known as the TME. TME is a multifaceted and interconnected network containing various cells and components, including extracellular matrix, lymphocytes, tumor-associated macrophages, dendritic cells, growth factors, cytokines, chemokines, natural killer cells, myeloid-derived suppressor cells (Fig. 1). TME affects not only the therapeutic effect of primary tumor treatments but also the evolution and advancement of tumor metastasis^5^. Within the TME, TAMs stand out as the most critical component, constituting approximately 50% of the tumor tissue’s weight^6^. TAMs originate from two different lineages, tissue-resident macrophage-derived and monocyte-derived. During the initial phases of tumor development, tissue-resident macrophages accumulate and distribute around tumor tissue to induce regulatory T-cell responses, promote tumor cell immune escape, provoke epithelial-mesenchymal transition (EMT), and enhance tumor cell infiltration and spread^7^. Numerous cytokines and chemotactic proteins, generated by adult hematopoietic stem cells within the circulatory system, attract monocyte-derived macrophages into the TME^7,8^. Subsequently, multiple stimuli promote the differentiation of these monocyte-derived macrophages into TAMs. Interleukin (IL)−1β, C-C motif ligand (CCL) 2, vascular endothelial growth factor (VEGF), and stromal cell-derived factor (SDF)−1α produced in tumors recruit pro-angiogenic macrophages to the tumor organs. Schmid et al. ^9^ demonstrated that the interaction of integrin-α4β1 with talin and paxillin, typically enhanced by IL-1β and SDF-1α-induced signaling, can be obstructed by leveraging antagonists against integrin-α4β1. While the exact timing of the conversion of recruited monocytes into TAMs has not been clarified, there is robust evidence supporting tissue-mediated alterations in the transcriptional profiles of these recruited monocytes^10^.

### The plasticity and polarization regulation of TAMs and their nomenclature

In reality TAMs exist as a continuum with high levels of plasticity and their expression types can often coexist or change as the tumor progresses. Ali N. Chamseddine et al. summarized the plasticity and diversity of TAMs and proposed the concept of TAM polarization as a continuous, dynamic polarization^11^. Although the nature, mechanism and nomenclature of TAM polarization still need further exploration, the artificial description of M1-like TAMs and M2-like TAMs^12^, located at distinct opposite ends within the continuous dynamic polarization axis of the TAMs and derived from summarization based on in vitro experiments, is unanimously confirmed^13^.

At present, the hypothesis of tumor suppressor M1-tumor promoter M2 proposed by Albert Mantovani^14^ is still the most commonly used model for studying TAM heterogeneity, although it oversimplifies the true phenotype of TAMs. However, recent in-depth studies on macrophages have revealed significant differences in structural design, operational capabilities, and expression of cellular surface identifiers among tissue-resident macrophages (TRMs) across various organs^15^. Furthermore, genes linked to M1-like and M2-like TAM profiles are expressed simultaneously in almost all types of cancer macrophage subgroups^16^. Recently, employing Single-Cell Regulatory Network Inference and Clustering (SCENIC) analysis^17^, researchers have discerned five specific TAM subgroups in various cancers. These subgroups are named *HES1* TAMs, *C1Q*^hi^ TAMs, *TREM2* TAMs, *IL4I1* TAMs, and proliferative TAMs. Utilizing single-cell genomics, Ma et al. ^18^ classified TAMs into seven subgroups based on characteristic genes, enriched pathways, and predicted functions. These subgroups are named IFN-TAMs, Reg-TAMs, Inflam-TAMs, LA-TAMs, Angio-TAMs, RTM-TAMs, and Prolif-TAMs.

Although M2-like macrophages have been thought to have anti-inflammatory functions while M1-like macrophages have pro-inflammatory functions, the reality of their behavior may be more complex. The characteristics and operational roles of macrophages are subject to change in response to environmental variations, and their roles can differ across various pathological states. Consequently, while present research tends to divide macrophages into categories based on their inflammation-related roles, with one being pro-inflammatory (often referred to as M1) and the other being anti-inflammatory (commonly known as M2), this binary division might not fully capture their biological diversity.

### The phenotypes and functions of TAMs

**Fig. 2:**
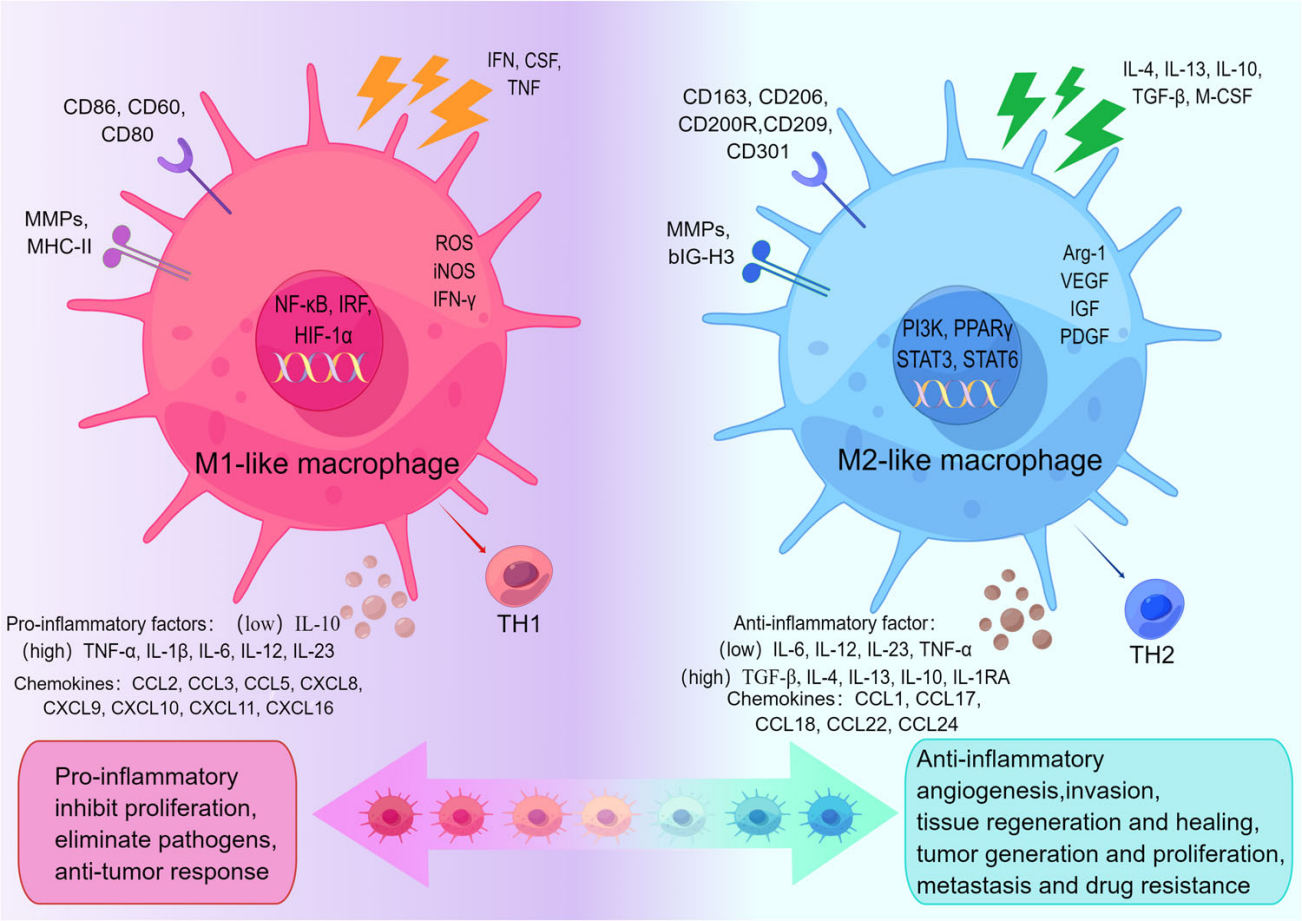
M1-like and M2-like TAMs are shown at opposite ends of a continuous dynamic TAM polarization axis, illustrating their unique roles in the TME. Both M1-like TAMs and M2-like TAMs have specific cell surface markers and functional factors. M1-like TAMs in the TME undertake roles in promoting inflammation, inhibiting proliferation, eliminating pathogens, and anti-tumor responses. M2-like TAMs in the TME are involved in anti-inflammatory activities, promoting angiogenesis, influencing tissue regeneration and healing, and fostering tumor generation, proliferation, metastasis, and drug resistance. (By Figdraw).

The M1-like TAMs and M2-like TAMs are located at the two ends of the continuous dynamic TAM polarization axis, each possessing unique cell surface markers and functional factors, and play different roles in the TME (Fig. 2).

The M1-like TAMs have pro-inflammatory properties. M1-like macrophages are stimulated by cytokines like interferon (IFN), colony-stimulating factor (CSF), tumor necrosis factor (TNF). Furthermore, lipopolysaccharides (LPS) have the capacity to interact with and engage the toll-like receptor (TLR)4 located on the macrophage surface and promote M1-like macrophage polarization by acting on nuclear factor kappa-B (NF-κB) and interferon regulatory factor 3 (IRF3). Strongly presenting antigens and secreting many pro-inflammatory cytokines are two traits of M1-like macrophages. M1-like macrophages secrete an extensive number of co-stimulatory molecules like cluster of differentiation (CD)86, CD60, and CD80, along with pro-inflammatory biomarkers including TNF-α, IL-1β, IL-6, IL-12, IL-23. They also highly express major histocompatibility complex (MHC) II molecules. Notably, they do express IL-10, although at a lower level. M1-like macrophages release matrix metalloproteinases (MMPs) including MMP1, MMP2, MMP7, MMP9, and MMP12. These enzymes are specialized in breaking down extracellular matrix (ECM) constituents. M1-like macrophages generate chemokines like CCL2, CCL3, CCL5, C-X-C motif chemokine ligand (CXCL) 8, CXCL9, CXCL10, CXCL11, CXCL16. Also, they release IFN-γ, inducible nitric oxide synthase (iNOS), and reactive oxygen species (ROS). M1-like macrophages activate a strong T-helper1(Th1)-type immune response by releasing these inflammatory mediators to promote an inflammatory response that inhibits cell proliferation and kills pathogens and tumor cells in the human body, thereby exerting antitumor effects^8,19–21^.

The M2-like TAMs, which present antigens poorly, are anti-inflammatory, unlike the M1 phenotype. There is no specific procedure that must be used to initiate the activation of M2-like macrophages. The primary activation of M2-like macrophages is caused by triggering cytokines, which include transforming growth factor (TGF)-β, IL-4, IL-13, IL-10, and macrophage colony-stimulating factor (M-CSF)^22,23^. M2-like macrophages highly express CD163, CD206, CD200R, CD209, CD301 and chemokines like CCL1, CCL17, CCL18, CCL22, and CCL24. They release numerous anti-inflammatory factors, including TGF-β, IL-4, IL-13, IL-10, and IL-1RA. Additionally, M2-like TAMs express the inflammatory cytokines IL-6, IL-12, IL-23 and TNF-α at lower levels^24^. M2-like macrophages highly express MMPs and autocrine ECM components such as fibronectin, betaig-h3 (BIG-H3), ECM cross-linking enzymes, transglutaminase and bone bridging proteins^25^, thus participating in cell adhesion. M2-like macrophages promote Arginase (Arg)−1 and VEGF expression, which are involved in the biosynthesis of proline and polyamines. Proline promotes the construction of ECM, and polyamines are involved in cell proliferation^26^. Other factors secreted by M2-like macrophages that promote cell proliferation, such as platelet-derived growth factor (PDGF) and insulin-like growth factor (IGF), are involved in angiogenesis^27^. M2-like macrophages release immunosuppressive chemicals to block the Th1-type immune activity as well as boost the Th2-type immune activity. This activity reduces the control over inflammatory reactions while promoting tumor cell growth, drug resistance, angiogenesis, and tissue healing^28,29^.

Sica et al.^30^ summarized and proposed the theory that TAMs were dominated by M1 phenotype in the early stage of tumor. As the tumor developed, the TME underwent changes that progressively led to the transformation of TAMs from the M1 to the M2 phenotype. Therefore, TAMs in most tumors present a dominant population with the M2 phenotype. TME in these tumors are generally immunosuppressive^31^, affecting tumor progression and chemotherapy resistance.

Moreover, in response to particular triggers and changes in gene expression within the TME, M2-like macrophages can be classified toward multiple subsets, namely M2a, M2b, M2c, and M2d^32,33^. Each of the subsets exhibits its own unique properties (Table 1). M2a macrophages^34^ receive their primary activation signals from the cytokines IL-4 and IL-13, which are produced by Th2 cells. These macrophages contribute to anti-inflammatory processes through their production of IL-10 and TGF-β. Furthermore, they are instrumental in allergy, wound healing, cell proliferation, profibrotic. Within the TME, they contribute to the advancement of tumors by enhancing the growth and infiltrative behavior of tumor cell and angiogenesis. Activation of M2b macrophages^35^ is generally triggered by a synergistic interaction between complexes involving the immune system and ligands of Toll-like receptors. They are known to generate a significant quantity of cytokines, including IL-10 and TNF-α, thus promoting inflammatory responses and participating in immune regulation. Within the TME, activated M2b macrophages secrete IL-6, which in turn activates Th2 cells, culminating in the creation of a pro-inflammatory milieu conducive to tumor progression. The activation of M2c macrophages predominantly occurs in response to IL-10, TGF-β, or glucocorticoids. They actively contribute to modulating inflammatory reactions by the release of anti-inflammatory molecules. Additionally, they are instrumental in the development of pathological fibrosis and the healing of wounds. They also possess immunoregulatory functions, capable of influencing immune responses by releasing specific factors and engaging in interactions with other immune cells. M2d macrophages^32,33^ represent a relatively recently identified subtype that is triggered by TLR agonists and IL-6. They are known to modulate the local inflammatory environment by releasing specific factors that possess both pro-inflammatory and anti-inflammatory properties. M2d macrophages support tumor cell immune evasion via emitting immunosuppressive substances like IL-10 and PD-1, inhibiting normal immune responses. By encouraging tumor growth, invasion, and angiogenesis, they aid in the advancement of tumors.

**Table 1:** Comparison of markers and biological functions among different macrophage phenotypes

Phenotypes	Functions	Stimulator factors	Markers	Excretion	References
M1	Tumor resistance, pro-inflammatory, inhibit proliferation, eliminate pathogens	IFN-*γ*, LPS, GM-CSF, TNF-α, iNOS, ROS	IL-12 (high)/IL-10(low), CD80, CD86, CD60, MMPs, MHCII	CCL2, CCL3, CCL5, CXCL8, CXCL9, IL-1β, IL-6, IL-23	^32^
M2a	Allergy, profibrotic, cell proliferation, anti-inflammatory, wound healing, tumor progression	IL-4, IL-10, IL-13, (PPAR-γ)	CD11b, CD45, CD86, CD14, CD206, CD163, CD209, IL-1R, Dectin-1, YM1, RELMα, IGF1, DCIR, Stabilin 1, Factor XIII-A, Ly6C, TREM-2, DC-SIGN, MHCII, Fizz1, Arg-1, YM1/2	TGF-β, IL-4, IL-10, VEGF, CCL1, CCL17, CCL18, CCL22, TNF-α, IGF, EPGF	^32–35^
M2b	Th2 activation, immunoregulation, inflammation, tumor progression	IL-1β, LPS, TLR	CD163, CD14, CD86, MHC-II, IL-10 (high), IL-12 (low), IL-6R	TNF-α, IL-1, IL-6, IL-10, CCL1, SPHK1, LIGHT	^32,35^
M2c	Immunoregulation, inflammatory response, wound healing, fibrosis	IL-6, IL-10, glucocorticoids, TGF-β, TNF-α	CD163, CD206, CD14, CD16, CD86, CXCR4, MerTK, TLR-1、TLR-8	IL-10, TGF-β, CCL16, CCL18, CXCL13	^32,35^
M2d	Tumor proliferation, invasion, and angiogenesis, immune suppression	IL-6, TLR, Regadenoson, LPS	IL-10 (high), IL-12 (low), VEGF, TNF-α (low), CD163, CD14, CD86	IL-10, IL-6, TGF-β, VEGF, CCL18, M-CSF	^32^

### The regulation mechanisms of M1-like /M2-like macrophages polarization

Current studies have shown that several cytokines and signaling pathways within TME influence M1/M2 polarization. Abnormalities of the IκB kinase β (IKKβ)/ NF-κB pathway may induce TAM polarization^36^. The process by which IL-4 binds to its receptor can promote signal transducer and activator of transcription (STAT)6 phosphorylation, inducing M2-like macrophage polarization via the janus protein tyrosine kinase (JAK)/ STAT6 signaling pathway. Concurrently, phosphorylated STAT6 can bind to krüppel-like factor 4 (KLF)4 and peroxisome proliferator-activated receptor-γ (PPAR-γ) to promote this polarization^37^. When IL-4’s interaction to its associated receptor is blocked, M2-like macrophages are induced to polarize to M1-like macrophages^38^. A variety of signals, including IL-4, TGF-β, IL-10, and bone morphogenetic protein-7 (BMP-7), promote M2 polarization through the phosphatidylinositol 3 kinase (PI3K)/ protein kinase B (Akt) signaling pathway^39^. Long non-coding RNA (LncRNA)-Xist knockdown within M1-like macrophages, or overexpression of miR-101 inhibits CCAAT/enhancer binding protein α (C/EBPα) and KLF6 production, which induces polarization from M1 to M2 phenotype^40^. Antitumor therapeutic strategies regulating M1/M2 polarization are promising due to the superior plasticity of TAMs.

## M2-like TAMs have involvement in promoting multidrug resistance in tumor cells

Researchers have shown via ongoing studies on M2-like macrophages that their potential to interact with tumor cells in a direct or indirect way, influencing anticancer medication therapeutic tolerance (Table 2) like chemotherapy, targeted therapy, and immunotherapy^41^. The possible regulatory mechanisms by which M2-like macrophages affect tumor drug resistance are increasingly elucidated through numerous studies.

**Table 2:** Mechanisms of inducing tumor drug resistance through M2-like TAMs

	Target	Host cell	Strategy	Mechanism	Refs
Signaling pathway	PI3K/ Akt	MCF7	Tamoxifen	Activation of PI3K/Akt/mTOR signaling by TAM-secreted CCL2 promotes the TME endocrine resistance feedback loop	^45^
		MCF7	Tamoxifen	Activation of breast cancer cells via EGFR/PI3K/Akt signaling by feedback upregulation of SGLT1	^46^
	JAK/ STAT	BT549, T47D	Paclitaxel	Modulation of IL-10/STAT3/bcl-2 signaling pathway	^49^
		MKN45	5-FU	Secretion of CCL8 to activate JAK1/STAT3 signaling phosphorylation	^50^
	Jagged1/Notch	MCF7	Aromatase inhibitor	Reprogramming TAMs through high expression of the Jagged1-Notch pathway	^59^
	NF-κB	TFK-1	Gemcitabine	M2-like TAMs-derived tgf-β1 induces EMT and drug resistance in CCA cells via the aPKCι/NF-κB signaling pathway	^63^
	Hippo	GBM	ICI	Promotion of promotes M2 polarization by SOH	^66^
Exosome	miR-21	MFC, MGC-803	Cisplatin	Modulation of the transfer of PTEN/PI3K/Akt signaling between TAMs and cancer cells by M2-derived miR-21 via the M2-specific ApoE	^81^
		OVCAR3, HO-8910	Chemotherapy	M2-delivered miR-21 enhanced OCA resistance via PI3K/Akt signaling	^82^
	MSTRG.292666.16	H1975	Osimertinib	M2-derived MSTRG.292666.16 promoted osimertinib resistance by regulating the miR-6386-5p/MAPK8IP3 axis	^83^
	miR-155-5p	DLD1, HCT-8, HT-29, LoVo	5-FU	Activation of the IL-6R/STAT3/miR-204-5p signaling by miR-155-5p in TAMs through regulating C/EBPβ in CRC	^84^
	DLBCL-exo	OCI-LY1. OCI-LY3	Epirubicin	DLBCL-generated exosomes may promote M2 polarization through activating the GP130/STAT3 signaling pathway and highly expressing IL-10, CD206 and CD163 expression	^85^
	miR-588	SGC7901	Cisplatin	Stimulation of the NF-kB signaling pathway by miR-588 through partially targeting cylindromatosis in GC to prevent apoptosis	^86^
	MCF7-exo	MCF7/S, MCF7/DOC	Docetaxel	Exosomal delivery and the release of P-gp export the chemotherapeutic agents outside tumor cells	^88^
	miR-365	K989	Gemcitabine	Adoptive transfer of miR-365 in TAMs induced gemcitabine resistance	^89^
	miR-1246	HeyA8, Skov3ip1, A2780	Paclitaxel	miR-1246 actives P-gP by targeting Cav1/P-gP/PRPS2/M2-like macrophages signaling pathway to inhibit paclitaxel uptake and transport	^90^
	SOX2-OT	H1975	EGFR-TKIs	SOX2-OT, as a miRNA sponge, targeted miR-627-3p activity and upregulated Smads expression, thereby reprogramming TAMs	^93^
	SNHG7	H1299, SPC-A1	Docetaxel	SNHG7 induces PTEN downregulation iva recruiting CUL4A, thus stimulates the PI3K/Akt signaling pathway to induce autophagy and M2 polarization.	^94^
	lnc-TALC	LN229, GL261, HMC3, BV-2	Temozolomide	GBM-delivered lnc-TALC can bound to ENO1 to activate p38MAPK signaling, thus increasing C5/C5a secretion to promote M2 polarization	^96^
	LINC00337	MCF7,MDA-MB-231	Paclitaxel	Recruitment of M2-like TAMs by LINC00337 induces tumor development and chemoresistance	^98^
	HCG18	SW620	Cetuximab	Promotion of M2 polarization by HCG18 via the miR-365a-3p/FOXO1/CSF-1 axis	^99^
	MIR155HG	Caco2, HT29	Oxaliplatin	Acceleration of the CRC evolution by MIR155HG through modulating the miR-650/ANXA2 axis enhances oxaliplatin resistance	^101^
	CRNDE	MFC, SGC7901	Cisplatin	M2-delivered CRNDE inhibits PTEN ubiquitination to reduce the susceptibility of cisplatin	^101^
Cytokine	TNF-α	LM2	Chemotherapy	TNF-α accumulated heavily in the TME, promotes the upregulation of CXCL1 and CXCL2 through activating the NF-kβ pathway	^109^
		MDAMB231, 4T1, E0771	Bevacizumab	M2b TAMs promote tumor metastasis via TNF-α, and activate IDO1	^110^
		SMMC-7721	Anti-tumor drug	M2-like TAMs promote EMT and CSCs via the Wnt/β-catenin pathway	^111^
	Interleukin	HepG2, SMMC7721,	Oxaliplatin	Activation of CMA signaling pathway by M2-like TAMs via IL-17/IL-17R pathway	^116^
		MCF7	Doxorubicin	The polarization of M2-like TAMs enhances the IL-6 paracrine loop between TAMs and tumor cells	^117^
		M109, H1975, PC-9	Osimertinib	EGFR T790M-cis-L792F activated the JAK/STAT3 pathway to promote the M2 polarization	^118^
	Chemokine	HCT-8, HCT-116, SW620, SW480, DLD1 CT26, HT-29	ICI	Activation of p65/STAT3-CSN5-PD-L1 signaling by TAM-secreted CCL5 inhibits CD8 + T cell responses in tumor cells	^122^
		DLD1, HT29	5-FU	Secretion of CCL22 activates the EMT program, PI3K/Akt pathway and Caspase-mediated apoptosis	^123^
	IGF	SUIT2, MIA-PaCa-2	Gemcitabine	Activation of insulin/IGF1R survival signaling by M2-like TAMs or by IGF, which is modulated by M2, enhances the resistance to gemcitabine	^125^
TME	CCL5	DU145, PC-3	Paclitaxel, Doxorubicin	Activation of STAT3-related signaling pathway by TAM-secreted CCL5 upregulates Nanog	^128^
	Treg	CNE1, CNE2, 5-8 F	ICI	M2-like TAMs recruit mature Treg by secreting CCL22, CCL18 and promote the conversion of naive T cells to Treg by secreting TGF-β, IL-10	^130^
Proangiogenic factor	VEGF	U87-MG	Bevacizumab	Depletion of VEGF causes MIF downregulation and promotes M2 polarization	^137^
		LN229, U251	Temozolomide	Secretion of VEGF by hypoxic M2-like macrophages through activating the PI3K/Akt/Nrf2 pathway	^138^
	VEGF-A	LLC	Cyclophosphamide, Cisplatin	Secretion of VEGF-A by M2-like TAMs to promote VEGFR2 phosphorylation	^139^
		A549	Doxorubicin	M2-like macrophages promote VEGF-C and VEGFR3 expression thereby inhibiting p53 and PTEN expression	^140^
CSCs	GSC20,GSC267	GBM	ICI	secretion of GDEs to induce monocyte polarize into M2-like macrophages via STAT3 signaling	^144^
	CSC	Cal27	Vincristine	M2-like TAMs promote OSCC cells to produce csc-like cells and overexpress stemness-related genes	^149^
	GSC	GBM	Chemotherapy	M2-like macrophages promote CSCs to express stemness features by mediating PTN - PTPRZ1 paracrine signaling induction	^150^

### M2-like TAMs modulate signaling pathways to enhance tumor resistance

By producing and releasing mediators, M2-like macrophages enhance cancer cell drug resistance by regulating PI3K/Akt, JAK/STAT, mitogen-activated protein kinases (MAPK) and other related pathways.

The PI3K signaling pathway, an intracellular signaling system influenced by receptor tyrosine kinases, modulates numerous cell processes including growth, division, maturation, metabolism and apoptosis^42^. Recent studies have identified the overactive PI3K/Akt pathway as a major factor in driving tumor growth and the emergence of treatment resistance in tumors^43^. Clinical research has demonstrated the efficacy of inhibitors targeting the PI3K/Akt pathway in cancer. Researchers have found that the release of cytokines, chemokines and GFs by M2-like TAMs modulate tumor PI3K/Akt signaling and modify the TME to advance tumor growth, differentiation, invasion, and drug resistance^44^. TAMs in breast cancer tissues increase tumor cells’ capacity to resist apoptosis via the CCL2/PI3K/Akt/ mammalian target of rapamycin (mTOR) signaling pathway, as found by Li et al.^45^. This induced tumor cells to undergo autophagy, leading to tamoxifen resistance. M2-like macrophages activate cancerous breast cells via the epidermal growth factor receptor (EGFR)/PI3K/Akt signaling pathway. This stimulation leads to the feedback increase in Sodium-glucose Co-transporters (SGLT) 1 and modulates glycolysis, thereby promoting tamoxifen resistance and accelerating both in vitro and in vivo tumor growth^46^.

With over 50 cytokines and growth factors (GFs) known to induce downstream signaling, the JAK/STAT signaling pathway has been established to be a primary communication hub in cellular function. It influences several critical biological processes like immunological modulation, cell development, and apoptosis. The promotion of tumor development, metastasis, and drug resistance is significantly aided by the abnormal and persistent activation of JAK-STAT signaling pathway proteins^47^. It’s an effective target for treating tumors. With in-depth studies of M2-like macrophages, researchers found that tumor-derived factors can polarize macrophages via JAK/STAT activation. Furthermore, certain mediators associated with macrophages can activate JAK/STAT signaling within tumors, subsequently contributing to drug resistance in these tumors^48^. M2-like macrophages may reduce the efficacy of paclitaxel against breast cancer through the IL-10/STAT3/Bcl-2 cascade response, thereby inducing resistance of paclitaxel against breast carcinoma^49^. The Yes-associated protein (YAP)1 overexpress IL-3, which is secreted by gastric cancer (GC). This overexpression prompts a significant TAM polarization toward M2 phenotype, subsequently initiating a GLUT3-mediated glycolytic program. Concurrently, by highly expressing CCL8 and triggering activation of the JAK1/STAT3 pathway, polarized M2-like macrophages increase tumor cells’ resistance to 5-fluorouracil (5-FU)^50^.

The MAPK signaling pathway relays signals via a highly conserved three-step kinase cascade: Initially, MAP kinase kinase kinase (MKKK) phosphorylates and activates MAP kinase kinase (MKK); subsequently, activated MKK phosphorylates and activates MAP kinase (MAPK). Four subfamilies of the evolutionarily conserved serine-threonine kinases—namely, MAPK- extracellular signal-regulated kinase (ERK), p38MAPK, c-Jun N-terminal kinase (JNK), and big mitogen-activated protein kinase 1 (BMK1) — represent distinct MAPK pathways^51^. As a crucial signaling pathway within the eukaryotic signaling network, the MAPK signaling pathway is essential for cell growth, differentiation, apoptosis, and stress response.

According to certain theories, the MAPK pathway alters the TME to cause the proliferation and invasion of human malignancies^52,53^, as well as polarizes macrophages into M2 phenotypes^54^. However, it remains unclear if M2-like TAMs may induce drug resistance in tumors by activating the MAPK pathway, warranting further exploration by researchers.

Various solid tumors and hematologic malignancies have abnormal expression of the Notch pathway^55^. Its role in TAM was summarized by Tanapat Palaga et al.^56^, who pointed out that different roles for Notch signaling exist in promoting or inhibiting tumor progression, depending on the specific TAM context. Targeting Notch signaling in different macrophages might offer varied therapeutic approaches to modulate host antitumor immunity. If Notch signaling in TAMs plays a tumor-promoting role by mediating macrophage polarization and modulating the TME, its inhibition might address tumor drug resistance^57,58^. According to Liu et al.^59^, when the Jagged1/Notch pathway is activated in a malignant breast tumor, TAMs polarize strongly toward M2 phenotype, subsequently increasing resistance to aromatase inhibitors. Conversely, if TAMs depend on Notch signaling to differentiate into inflammatory antitumor macrophages, stimulating this particular Notch pathway could be vital to achieve tumor-suppressive outcomes^60^.

The transcription factor NF-κB is crucial for inflammatory reactions and serves as a key molecule that connects cancer and chronic inflammation. The functions of it are tightly modulated through various processes. The progression of many solid tumors involves abnormal NF-κB pathway activation^61^. It is now known that through controlling the NF-κB pathway, macrophages may foster tumor progression and resistance^62^. In cholangiocarcinoma, M2-like TAMs induce EMT and gemcitabine resistance in tumor cells through the atypical protein kinase Cι (aPKCι)/NF-κB signaling pathway^63^.

By controlling cell growth and stem cell self-renewal, the evolutionarily conserved mechanism known as Hippo signaling regulates organ growth. Dysregulation of this route may contribute to cancer development^64^. Chen et al.^65^ observed that lung adenocarcinoma (LUAD) cells received exosomal LINC00273 from M2-like TAMs. This triggered the ubiquitination of large tumor suppressor 2 (LATS2), deactivating the Hippo pathway and subsequently activating the YAP protein downstream of LATS2. This sequence of events encouraged the incorporation of miR-19b-3p into LUAD cell-derived exosomes, exacerbating the malignant behavior of LUAD cells. In their conclusion, YAP is a viable target in therapies aimed at targeting tumors. Kim EH’s analysis^66^ used the Cancer Genome Atlas (TCGA) database to focus on glioblastoma specimens.

This analysis aimed to predict and validate differences between the silenced Hippo pathway (SOH) and the active Hippo pathway (AH) groups. The findings revealed that M2-like macrophages were up-regulated in the SOH group. This up-regulation was linked to a bad prognosis for glioblastoma multiforme (GBM), indicating the possibility that SOH might cause GBM to become immunity resistant.

### TME-derived exosomes target M2-like TAMs to enhance tumor resistance

Exosomes function as cell signal transducers. Almost all types of cells produce exosomes; however, their quantities vary and are cell-specific^67^. In macrophage biology, we tend to refer to them broadly as macrophage-derived extracellular vesicles (Mp-EVs). Mp-EVs, particularly those with sizes ranging from 40 to 160 nm (average about 100 nm)^68^, act as shuttle carriers. They transport various kinds of bioactive materials, including proteins, metabolites and nucleic acids (DNA, mRNA, lncRNA, miRNA) between diverse cells within the TME. Tumor-derived exosomes can induce stromal cell differentiation into tumor-associated cells, transforming the antitumor environment into a pro-tumor one, and can confer tumor resistance and promote tumor metastasis by triggering EMT execution^69^.

#### Exosomal miRNAs

A short non-coding RNA, called small molecule ribonucleic acid (miRNA), controls the expression of several target genes. This modulates critical biological processes like cell invasion, differentiation, and medication resistance^70,71^. Exosomal miRNAs can convert macrophages into either the M2 or M1 type^72^. Unlike plasma miRNA, exosomal miRNA is enveloped by a lipid bilayer, which protects it from degradation by RNA hydrolases in the extracellular milieu, thus enhancing its stability^73^. In the TME, exosomal miRNAs promote tumor angiogenesis, cell migration^74^, invasion, metastasis^75,76^ and drug resistance^77–79^ by reprogramming M2-like TAMs. Anticancer medication resistance is strongly associated with abnormal expression of PI3K/Akt, STAT3, MAPK, and other signaling pathways^80^. Exosomes from TAMs can modify these signaling pathways to modulate therapeutic resistance. In GC^81^, exosomal miRNA-21 generated by M2-like TAMs regulates the transfer of phosphatase and tensin homolog (PTEN)/PI3K/Akt signaling between TAMs and cancer cells through the M2-specific apolipoprotein E (ApoE). This enhances GC cells’ resistance to cisplatin (DDP). In ovarian cancer (OCA), miRNA-21 not only promotes M2-like TAMs polarization but also, when delivered by M2-like TAMs through PI3K/Akt pathway, enhances chemotherapeutic agent resistance^82^. The regulation of the MSTRG.292666.16/miR-6386-5p/MAPK8IP3 axis by exosomes derived from M2-like macrophages may potentially contribute to the development of resistance to Osimertinib in patients with non-small cell lung cancer (NSCLC)^83^. MiR-155-5p in TAMs^84^ can activate the IL-6R/STAT3/miR-204-5p pathway in colorectal cancer (CRC) to induce tumor chemoresistance by regulating C/EBPβ. Ling et al.^85^ proved that exosomes produced from large diffuse B-cell lymphoma (DLBCL) might inhibit epirubicin-induced apoptosis in DLBCL cells, stimulate the GP130/STAT3 pathway, upregulate IL-10, CD206, and CD163 expression, and polarize TAMs toward M2 phenotype in order to enhance epirubicin tolerance in DBCL. M2-derived Exosomes may target tumor suppressor elements, curtail cell apoptosis, accelerate tumor expansion, and lead to chemotherapy resistance. For example, exosomes can successfully increase the tumor cell resistance to DDP by partially targeting cylindromatosis in GC cells, stimulating the NF-kB signaling pathway, and preventing apoptosis^86^.

In addition, exosomes from TAMs can not only alter various signaling pathways to modulate therapeutic resistance, but also modulate drug resistance by transferring chemotherapy drugs outside tumor cells. Another significant contributor to treatment resistance in tumor cells is the horizontal transfer of exosomes harboring drug efflux pumps^87^. P-glycoprotein (P-gp), commonly referred to the multidrug resistance gene (MDR1), represents a well characterized transport protein involved in anticancer drug efflux. Exosomal delivery and the release of P-gp export chemotherapeutic agents out of tumor cells, leading to chemoresistance^88^. TAMs-derived exosomes inactivate the gemcitabine pool and promote gemcitabine resistance in pancreatic ductal adenocarcinoma (PDAC) cells via miR-365 transfer, upregulating nucleotide triphosphates and inducing cytidine deaminase in PDAC cells^89^. In OC, Pinar et al.^90^ found that miR-1246 activates P-gP by targeting Cav1/P-gP/PRPS2/M2-like macrophages signaling pathway to inhibit paclitaxel uptake and transport.

#### Exosomal lncRNAs

Long non-coding RNAs (lncRNAs) are non-coding RNAs exceeding 200 nucleotides in length and play pivotal roles in genomic expression and regulation. They are closely linked to several kinds of diseases, particularly tumor development, progression, and treatment resistance. Exosomal lncRNAs, originating from TAMs, influence the TME. They promote tumor proliferation, metastasis, and angiogenesis. Moreover, they contribute significantly to drug resistance and the establishment of an immunosuppressive microenvironment. They act on specific target cells through signaling between malignant cells and non-transformed cells^91,92^.

The NCI-H1975 line of NSCLC, characterized by EGFR mutations, releases exosomal SOX2-OT to macrophages. SOX2-OT, functioning like the miRNA sponge, targets and absorbs miR-627-3p to inhibit miR-627-3p activity and upregulate Smads expression, which in turn encourages M2 polarization of macrophages while inhibiting M1-like macrophages. Consequently, the induced M2-like macrophages enhance the resistance of H1975 cells to EGFR-tyrosine kinase inhibitors (TKIs)^93^. Exosomal SNHG7 downregulates PTEN by recruiting cullin 4 A (CUL4A), subsequently stimulating the PI3K/Akt pathway. Based on this, exosomal SNHG7 induces M2-like TAMs polarization and autophagy to enhance docetaxel resistance in LUAD cells^94^. Complement Component 5 (C5) is a complement system component and a cytokine involved in DNA damage repair^95^. C5 regulates M2-like TAMs polarization and reshapes the immunosuppressive GBM microenvironment. Li et al.^96^ found that lnc-TALC, which was transferred to microglia by GBM-derived exosomes, bound to ENO1 in microglia. This binding activated p38MAPK signaling, increase C5/C5a secretion, promoted M2-like macrophage polarization in microglia, and enhanced GBM cell tolerance against temozolomide treatment. This shows that the interaction between microglia and GBM cells via lnc-TALC can reduce the impact of chemotherapy. In addition, Li demonstrated that C5a-targeted immunotherapy significantly reduced lnc-TALC-mediated temozolomide resistance. From these findings, it can be concluded that novel therapeutic strategies for blocking the interaction between microglia and GBM cells via lnc-TALC have particular potential value. LINC00337 is a relatively novel lncRNA whose differential expression level is closely related to tumor resistance^97^. Xing et al.^98^ discovered that in breast cancer (BC), LINC00337 is markedly overexpressed. This overexpression fosters BC cell proliferation, migration, EMT, and resistance to PAX chemotherapy by driving M2-like macrophage polarization. HCG18 promotes M2-like TAMs polarization by impacting the miR-365a-3p/ forkhead box-1(FoxO1)/CSF-1 axis, which in turn enhances cetuximab resistance in CRC cells. This is a brand-new cetuximab resistance mechanism in CRC^99^. The lncRNA MIR155HG was observed to compete with annexin A2 (ANXA2), a protein that promotes tumor progression, to bind miRNA and promote the expression of ANXA2 to aid in the formation of glioblastoma^100^. Zhou et al.^101^ found that MIR155HG specifically targeted ANXA2 in addition to competing with it to bind miR-650. M2-like macrophage polarization was suppressed by the knockdown of MIR155HG or ANXA2. This led to a decline in proliferation, migration, invasion, and oxaliplatin resistance of CRC cells. Based on these observations, Zhou concluded that MIR155HG could accelerate the evolution of CRC and enhance oxaliplatin resistance via altering the miR-650/ANXA2 axis in CRC. According to Xin et al.^100^. lncRNA CRNDE was overexpressed in both cancer tissues and TAMs of GC patients. They found that the exosome-mediated transfer of CRNDE from M2-like macrophages to GC cells could inhibit PTEN expression, thereby reducing the susceptibility of GC cells to DDP. Silencing lncRNA CRNDE expression in M2 exosomes effectively reversed the DDP resistance in GC cells induced by M2 exosomes.

### M2-like TAMs modulate cytokines to enhance tumor resistance

Cytokines are small molecule peptides, proteins, or glycoproteins synthesized and secreted by various cells^102^. By binding to receptors on target cell membranes, cytokines transmit signals into the cell’s interior, orchestrating numerous kinds of biological functions, including immune modulation, inflammation, and tissue repair^103^. Cytokines can be categorized into the following groups based on their many primary functions: (1) ILs are biomolecules responsible for transmitting immunomodulatory information between leukocytes. Among cytokines in immunology, ILs are the most prevalent and significant; (2) CSFs are cytokines that selectively stimulate the hematopoietic progenitor cell proliferation in vivo as well as in vitro, causing them to differentiate and form colonies of specific cell lineages. The colony-stimulating factors are named based on their range of action as G-CSF, M-CSF, GM-CSF; (3) There are approximately 7 different forms of IFNs: IFN-α, IFN-β, IFN-γ, IFN-λ, IFN-ε, IFN-κ, and IFN-ω. They are created by leukocytes, fibroblasts, and activated T cells under the stimulation of virus, mitogen or double-stranded RNA, respectively. They play crucial roles in antitumor, antiviral, and immunomodulatory functions^104^; (4) The TNF superfamily comprises numerous receptors and approximately 19 ligands. There are two mainly categories within the TNF superfamily, both of which induce necrosis in tumor tissues and possess tumoricidal activity: TNF-α, produced by monocyte-macrophages, and TNF-β, produced by activated T cells; (5) TGF-family, primarily TGF and BMP, are created by multiple cells; (6) Growth factor (GF), such as VEGF, PDGF, IGF, TGF-α,epidermal growth factor (EGF), fibroblast growth factor (FGF), hepatocyte growth factor (HGF); (7) Chemokines, which have four cysteines located at highly conserved positions, are categorized as four distinct subfamilies according to the quantity and spacing of these highly conserved N-terminal cysteines family: CC, CXC, CX3C, and XC. M2-like macrophages secrete various cytokines that enhance multi-drug resistance in tumor cells.

#### TNF-α

TNF-α is a type II transmembrane protein that exerts multiple biological actions by binding to two distinct receptors. TNF-α, binding to tumor necrosis factor receptor (TNFR)−1, activates two distinct and complex signaling pathways: one that maintains cell survival and promotes inflammatory cytokine expression, and another that leads to apoptosis and necrosis. On the other hand, TNF-α binding to TNFR-2 typically initiates immune regulation, promotes epithelial cells expression and tissue regeneration^105^. As the most important inflammatory mediator in the tumor-associated inflammatory network^106^, TNF-α is essential for tumor signaling routes and immunity regulation. Specifically, TNF-α modulates tumor development, invasion, metastasis, acquired drug resistance and the induction of adaptive and innate immune responses^107^. TNF-α buildup in TME might mediate chemotherapy resistance in tumor patients undergoing chemotherapy, according to recent research^108^. Through activation of the NF-kβ pathway, TNF-α accumulates heavily in the TME, thereby promoting the CXCL1 and CXCL2 upregulation. These two chemokines play pivotal roles in mediate metastasis and chemoresistance^109^. M2-like TAMs secrete TNF-α and other pro-tumor cytokines and activate indoleamine2,3-dioxygenase1 (IDO1) to enhance tumor cell resistance to bevacizumab^110^. M2-like macrophages induce EMT and enhance tumor resistance in hepatocellular carcinoma (HCC) by secreting TNF-α via the Wnt/β-catenin pathway^111^.

#### Interleukin family

Interleukin is a cytokine involved in regulating systemic inflammation and the immune system. It is generated by an extensive variety of cells throughout the organism and can act on many different types of cells. At least 40 ILs have been identified and are named IL-1 to IL-40^112^. IL significantly influences cancer growth, development, angiogenesis, metastasis, and antitumor drug sensitivity. It serves as a critical mediator for information transfer between innate and adaptive immune cells as well as non-immune cells and tissues^113–115^. Guo et al.^116^ found that M2-like macrophages can activate the molecular chaperone-mediated autophagy (CMA) signaling pathway through the IL-17/IL-17R pathway, which decreases apoptosis sensitivity and induces oxaliplatin resistance in hepatocellular carcinoma cells. Li et al.^117^ discovered that in breast cancer, M2-like macrophages and tumor cells can enhance doxorubicin (DOX) resistance via the IL-6/IL-6R paracrine pathway. Sun et al.^118^ revealed that the EGFR T790M-cis-L792F induced the JAK/STAT3 pathway, promoting p-STAT3 (Tyr705) to bind specifically to the IL-4 promoter. This binding enhanced IL-4 expression and secretion, promoting the M2-like macrophage polarization, thereby enhancing resistance to osimertinib in NSCLC cells. In addition, they found that blocking STAT3/IL-4 inhibited the polarization of M2-like TAMs, overcoming the EGFR T790M-cis-L792F-induced resistance to osimertinib.

#### Chemokine family

Chemokines comprise roughly fifty species of small molecule secreted proteins. They can be classified into four groups: CC family, CXC family, CX3C family and XC family. Chemokines bind to G protein-coupled receptors with seven transmembrane structural domains on the cell surface^119^ to increase intracellular calcium levels and activate MAPK, PI3K and other signaling pathways, thereby stimulating cell migration, inducing cell adhesion, and promoting cell proliferation and differentiation^120^. Involved in the tumor immunological process and inducing tumor immune escape, chemokines are crucial elements of the TME^121^. CCL5 derived from TAMs^122^ can induce CSN5 expression through the p65/STAT3 pathway and promote stable Programmed Cell Death-Ligand 1 (PD-L1) expression in CRC cells, thus exerting immunosuppressive effects. CCL22 released by M2-like TAMs inhibits the 5-FU sensitivity of CRC by initiating EMT, stimulating the PI3K/Akt pathway, and inhibiting Caspase-mediated apoptosis^123^.

#### IGF

IGF-1, IGF-2, and IGF-3, in addition to their corresponding receptors IGF-1R, IGF-2R, and IGFBP, are the ligands that make up the IGF system. The structure and function of IGF bear similarities to insulin. IGF can promote cell proliferation, differentiation and cell secretion. It can also promote wound repair and bone anabolism, enhance glucose absorption and amino acids, and foster glycogen synthesis and lactate secretion. IGF is currently elevated in various kinds of cancers and directly correlates with tumor resistance^124^. Ireland et al.^125^ found that M2-like macrophages enhance gemcitabine resistance in pancreatic cancer by directly influencing IGF to initiate a paracrine pathway called the insulin/IGF1R survival signaling pathway. Their study showed that elevated levels of IGF-1/2 are expressed by BRCA-associated TAMs, which stimulates insulin/IGF-1 receptor signaling in tumor cells. The stimulation is linked to an advanced tumor stage and enhanced macrophage infiltration.

### M2-like TAMs modulate the immunological microenvironment facilitating immunosuppressive TME to enhance tumor resistance

The tumor immune microenvironment is a critical factor influencing tumor development and profoundly affects the antitumor therapy’s prognosis. In the tumor immune microenvironment, the ratio of M1-like/M2-like TAMs is crucial for regulating immune therapy responses. M1-like TAMs enhance anti-tumor immunity by producing cytokines like IFN-γ and TNF-α, which activate T cells and other immune cells, and by efficiently presenting antigens to assist T cells in targeting and eliminating tumor cells. In contrast, M2-like TAMs upregulate immunosuppressive surface proteins and related anti-inflammatory factors, including PD-L1, IL-10, TGF-β, and IL-4. These factors have the ability to impede the functionality of effector T cells and concurrently facilitate the development and activation of regulatory T cells (Tregs), thereby augmenting the immunosuppressive environment. Additionally, they can secrete factors that recruit Tregs, inhibit the proliferation of effector T cells and monocytes, and accelerate the depletion of effector T cells^31^. This action weakens the ability of T cells to suppress cancers within the TME and diminishes the anti-tumor immune response, thus aiding tumor cells in evading immune attack and promoting both tumor development and drug resistance. Therefore, a higher M1/M2 ratio reflects the dominance of pro-inflammatory M1-like TAMs, enhancing T cell immune responses to tumors and increasing the effectiveness of immunotherapy. In contrast, a lower M1/M2 ratio indicates a relative increase in immunosuppressive M2-like TAMs, promoting tumor immune escape, weakening immune responses, and facilitating tumor drug resistance. Furthermore, the presence of PD-1 in TAMs suppresses their phagocytic and cytotoxic activity against tumor cells, affects the polarization of TAMs, leading to an immunosuppressive M2 phenotype, enhances the immune escape mechanisms of tumor cells, and affects the tumor microenvironment^126,127^. Therefore, regulating the M1/M2 ratio and targeting M2-like TAMs to control the PD-1/PD-L1 pathway is particularly important in approaches for treating tumors and overcoming tumor drug resistance.

Ma et al.^128^ found that TAM-secreted CCL5 activates STAT3 signaling pathway to induce EMT and to upregulate the transcription factor Nanog. This process promotes resistance to chemotherapy drugs in prostate cancer. Roy et al.^129^ in their highly cited review on the therapeutic mechanism of TAM also mention that TAMs express PD-L1 and Cytotoxic T-lymphocyte-associated antigen (CTLA)−4 ligands, which block the T lymphocytes’ adaptive immune response and reduce the anticancer effects of immunotherapy by binding to PD-1 and CTLA-4 on the surface of T cells. M2-like macrophages secrete cytokines like CCL22 and CCL18 to recruit mature Treg. Additionally, they promote the conversion of naive T cells to Treg by secreting TGF-β and IL-10. These actions by M2-like macrophages enhance the development of tumor resistance^130^.

### M2-like TAMs modulate tumor angiogenesis to enhance tumor resistance

Through the regulation of MMPs, serine proteases and cathepsins, TAMs induce angiogenesis, degrade basement membranes, and secrete pro-angiogenic factors, cytokines and chemokines including VEGF, CXCL8, MMP7, MMP9 and MMP12, facilitating the formation of the tumor vascular networks^131^. VEGF is a highly bioactive functional glycoprotein that binds to two receptor tyrosine kinases (RTK): Vascular endothelial growth factor receptor (VEGFR)−1 and VEGFR-2^132^. Within the human body, various members of the VEGF family are expressed, including VEGF-A, VEGF-B, VEGF-C, VEGF-D, VEGF-E and Placental Growth Factor (PLGF)^133^. VEGF is specific in its action on vascular endothelial cells. It improves the permeability of postcapillary and small veins, causing plasma proteins to leak, and stimulating the production of neovascular growth factor. Endothelial cells originating from arterial, venous, and lymphatic systems are stimulated to proliferate, migrate, and form lumens^134^. VEGF acts as an essential regulator of tumor angiogenesis. The hypoxic conditions prevalent in solid tumors enhances VEGF production, thereby promoting tumor angiogenesis and subsequent tumor progression^135^. VEGF^136^ could promote M2-like macrophage polarization and synergize with M2-like macrophages to promote hypoperfused tumor angiogenesis. This process may prolong transvascular delivery of antitumor drugs to tumor tissues, thereby increasing the tumor tolerance to therapeutic agents. Such actions could affect the efficacy of antitumor therapy. Castro et al.^137^ observed that in glioblastoma, depletion of VEGF induced by bevacizumab downregulated macrophage migration inhibitory factor (MIF) at the tumor margin, leading to the proliferative expansion of M2-like TAMs. This process consequently promoted tumor growth and bevacizumab resistance. Zhang et al.^138^ have found that hypoxia promotes M2-like macrophage polarization in a transcription factor HIF-1α-dependent manner. In glioblastoma, hypoxic M2-like macrophages enhance VEGF secretion by stimulating the PI3K/Akt/Nrf2 pathway, a process that subsequently increases tumor resistance, angiogenesis, and cancer aggressiveness. The majority of macrophages present within tumors exhibit the M2 phenotype and secrete VEGF-A, a factor that promotes aberrant vascular development in tumor. This results in terminal vascular malformation, excessive branching, and increased vascular permeability, which collectively affect tumor hemodynamics and the transport of therapeutic agents. It was found that the removal of VEGF-A from macrophages inhibited the phosphorylation level of tumor VEGFR2, which led to a reversion to normal vascular development, and improved the sensitivity of Lewis Lung Carcinoma (LLC) cells to the cytotoxic drugs cyclophosphamide and cisplatin. This finding confirmed that macrophages could enhance resistance to chemotherapeutic agents by secreting VEGF-A^139^. In LUAD, M2-like TAMs downregulate the expression of p53 and PTEN while upregulating the expression of VEGF-C and VEGFR3, thereby attenuating apoptosis in cancer cells and inducing DOX resistance^140^.

### M2-like TAMs and CSCs modulate each other to enhance tumor resistance

Cancer Stem Cells (CSCs)^141^, a specialized type of tumor cells, possess the capacity to self-renew, exhibit extensive drug resistance, demonstrate sensitivity to differentiation signals, and maintain intratumoral homeostasis. CSC traits^142^ include indefinite self-renewal capacity, extensively drug resistance, and sensitivity to differentiation, are major contributing factors to the propensity of malignant tumors for chemoresistance. Current studies indicate that the interaction between CSCs and TAMs is crucial in the development of resistance to antitumor therapies^143^. CSCs can promote tumor drug resistance by inducing macrophage polarization toward the M2 phenotype. For example, GSC-derived exosomes (GDEs), which were secreted by Glioblastoma Stem Cells (GSCs), through the release of various biological factors, mediate the STAT3 immunosuppressive regulatory pathways^144^, inducing monocyte to polarize into M2-like TAMs, thereby promoting drug resistance. Concurrently, M2-like TAMs promote tumor proliferation, invasion, metastasis, and resistance by stimulating the stem cell properties of CSCs via the secretion of CSC-associated factors^145^. CD105 (Endoglin)^146^ and CD44^147,148^ are key cell surface molecules instrumental in tumor angiogenesis and defining the properties of CSCs, respectively. CD105 is involved in tumor development, inflammation, and the accumulation of CAFs (cancer-associated fibroblasts), while CD44 facilitates tumor cells adhesion, migration, and chemoresistance by interacting with hyaluronic acid. In Oral Squamous Cell Carcinoma (OSCC), M2-like TAMs promote the formation of CSC-like cells induced by OSCC, with overexpression of the Sox2, Oct4, and Nanog genes, leading to increased positive expression rates of CD44 and CD105, reducing OSCC apoptosis, enhancing cell migration, and promoting resistance to vincristine^149^. M2-like macrophages secrete Pleiotrophin (PTN), which interacts with the protein tyrosine phosphatase receptor type Z1 (PTPRZ1) receptor on the surface of CSCs. This binding stimulates the Fyn-Akt pathway, resulting in the sustained expression of stemness characteristics in CSCs and enhancing chemoresistance in OSCC cells^150^.

## Targeting M2-like TAMs to overcome tumor resistance

**Fig. 3:**
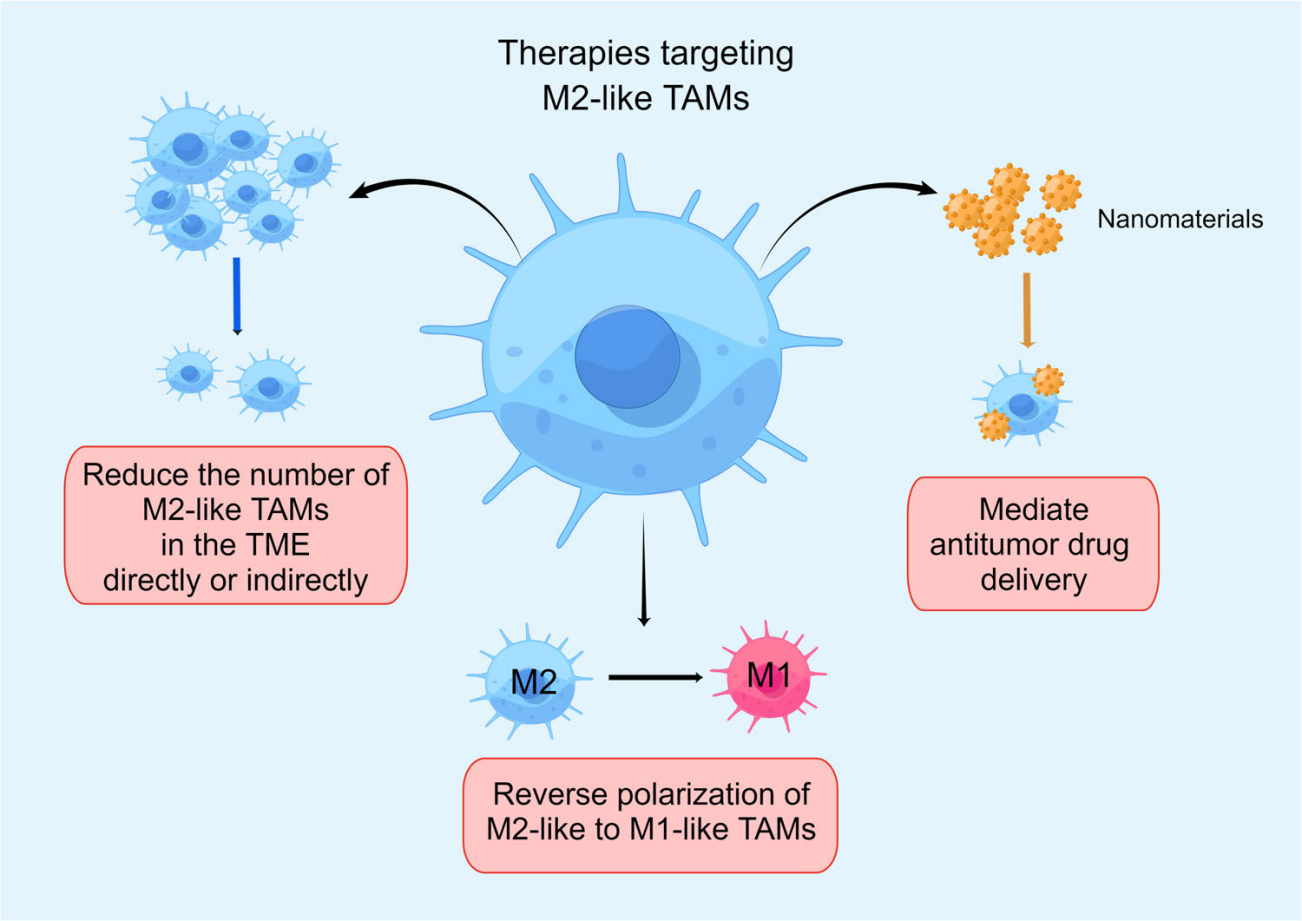
Current major antitumor approaches targeting M2-like TAMs to overcome tumor drug resistance. These approaches mainly encompass three aspects: reducing the number of M2-like TAMs in the TME through direct or indirect methods, thereby diminishing their role in promoting tumor growth; using M2-like TAMs as antitumor drug delivery mediums; and inducing the re-polarization of M2-like TAMs to the M1 phenotype, thus restoring their anti-tumor activity and enhancing the immune response in the TME. These strategies collectively form a comprehensive therapeutic approach to overcoming tumor drug resistance by targeting M2 TAMs. (By Figdraw).

Considering M2-like TAMs’ critical role in tumor drug resistance, the search for therapeutic approaches targeting these cells has emerged as a critical area of focus in anticancer therapy research. Currently, the main antitumor strategies based on M2-like macrophages (Tables 3, 4) include: (1) Directly or indirectly reducing the number of M2-like macrophages in TME. (2) Using M2-like macrophages as antitumor drug delivery mediums. (3) Repolarizing M2-like macrophages to the M1 phenotype. Based on these antitumor drug resistance strategies targeting M2-like TAMs, researchers have carried out numerous experimental experiments in both preclinical and clinical settings (Fig. 3).

**Table 3:** Antitumor nanodrug research targeting M2-like TAMs.

Strategy	Therapeutic agent	Tumor model	Therapeutic mechanism	Refs
NanoMnSor	sorafenib	O.T. mouse model of HCC	Co-deliver of oxygen-producing manganese dioxide (MnO2) and sorafenib to HCC to reduce the hypoxia-induced tumor infiltration of TAMs	^170^
a nanoliposome-loaded C6-ceremide (LipC6)	ceramide	O.T. mouse model of HCC	Activation of antitumor immunity by inducing TAMs reprogramming through regulating ROS signaling.	^171^
nanoparticles/bacteria complex (Ec-PR848)	Resiquimod (R848)	O.T. 4T1 mouse model of breast cancer	Reprogramming of TAMs by activating TRL7/8	^172^
a TAM-targeting probe consisting of CD206 antibody coupled to near-infrared phthalocyanine dye (IRD-αCD206)	sorafenib	M.T. 4T1 mouse model of breast cancer	Depletion of TAMs by phototherapy	^173^

**Table 4:** Clinical study on M2-like TAMs treatment strategies to overcome tumor drug resistance.

	Intervention	Statustumor type	Status	gov identifier	Refs
Reduce M2-like TAMs in TME					
JAK inhibitor	Ruxolitinib	Relapsed/refractory multiple myeloma patients	Completed	NCT03311854	^155^
CSF-1R inhibitor	Pexidartinib	Unresectable sarcoma and MPNST	Completed	NCT02584647	^151^
	Emactuzumab	Advanced/metastatic solid tumors	Completed	NCT01494688	^152^
	AMG 820	Advanced solid tumors	Completed	NCT02713529	^196^
	Cabiralizumab	Melanoma, RCC, or NSCLC resistant to anti-PD-1/PD-L1	Completed	NCT03502330	^197^
CCR5 inhibitor	Maraviroc	refractory MMRp/MSS metastatic CRC	Ongoing	NCT03274804	^152^
TAM inhibitor	Sitravatinib	Advanced clear cell renal cell carcinoma	Completed	NCT03015740	^198^
VEGFR2 inhibitor	Anlotinib	Advanced NSCLC	Completed	NCT03628521	^199^
Repolarize M2-like to M1-like TAMs					
CSF-1R inhibitor	Pexidartinib	Advanced, treatment refractory solid tumors	Completed	NCT01525602	^175^
	ARRY-382	Advanced solid tumors	Completed	NCT02880371	^200^
	BLZ945	Advanced solid tumors	Ongoing	NCT02829723	^175^
	LY3022855	Advanced solid tumors	Completed	NCT02718911	^201^
CSF-1 inhibitor	Lacnotuzumab	Advanced triple-negative breast cancer	Not yet recruiting	NCT02435680	^202^
	PD-0360324	Locally advanced or metastatic solid tumors	Ongoing	NCT02554812	^175^
BRD4 inhibitor	AZD5153	relapsed/refractory solid tumors or lymphoma	Completed	NCT03205176	^203^
CD47-SIRPα inhibitor	Evorpacept	HNSCC	Completed	NCT03013218	^178^
CD40 agonist	APX005M	Melanoma, NSCLC, or RCC	Completed	NCT03502330	^197^
	SEA-CD40	Advanced solid tumors and lymphomas	Completed	NCT02376699.	^181^
PI3Kγ inhibitor	IPI-549	Advanced solid tumors	Completed	NCT02637531	^204^
Src inhibitor	Dasatinib	NSCLC	Ongoing	NCT00826449	^205^
HDAC inhibitor	Tucidinostat	intermediate- and high-risk early-stage ENKTCL	Completed	NCT04511351	^206^
STAT 3 inhibitors	Danvatirsen	Advanced solid tumors	Completed	NCT03394144	^207^
TGFβ inhibitor	Galunisertib	Unresectable pancreatic cancer	Completed	NCT02734160	^208^
TLR agonist	Vidutolimod	Metastatic melanoma	Completed	NCT03084640	^209^

### Reduce the number of M2-like TAMs in the TME directly or indirectly

In order to recruit macrophages into the TME, tumor cells secrete various chemokines, including CSF-1, CCL2, CCL5, CX3CL1, and CXCL12. Once within the TME, macrophages are subject to modification by various tumor cell-released cytokines, metabolites, and exosomes, which change the activity of TAMs and promote their polarization. Thus, decreasing TAMs recruitment can indirectly reduce M2-like macrophages in the TME, thereby helping to inhibit tumor progression and overcome tumor drug resistance.

Pexidartinib, a colony-stimulating factor 1 receptor (CSF-1R) inhibitor, is capable of being safely used with sirolimus to inhibit the growth of unresectable sarcoma and malignant peripheral nerve sheath tumors by decreasing the number of M2-like TAMs^151^. Emactuzumab, another CSF-1R inhibitor, whether used as a single agent or in combination with paclitaxel in patients with advanced/metastatic solid tumors, demonstrated immunosuppressive M2-like TAMs depletion in the TME. However, neither in isolation nor in combination with paclitaxel did it result in clinically relevant antitumor activity^152^. Unfortunately, this Phase I trial did not find clinically relevant antitumor activity of emactuzumab, although it did suggest potential therapeutic benefits that merit further investigation. Guan et al.^153^ found that CSF-1R inhibitors reduced TAMs recruitment by inhibiting the CXCL12/CXCR4 signaling axis, thereby resisting macrophages polarization and increasing the sensitivity of prostate cancer to docetaxel treatment. Yang et al.^154^ discovered that paclitaxel-resistant ovarian cancer cells significantly increased macrophage migration and upregulated M2-like macrophage marker expression via massive secretion of CCL2. Therefore, targeting the CCL2/CCR2 axis could inhibit macrophage chemotaxis, potentially improving paclitaxel sensitivity in ovarian cancer patients. The JAK1/2 inhibitor ruxolitinib (RUX) can overcome myeloma’s tolerance to the immunomodulatory drug lenalidomide^155^. Further studies revealed that Ruxolitinib overcame drug resistance by inhibiting M2-like macrophage polarization through the downregulation of TRIB1, MUC1, CD44, CXCL12, and CXCR4 within the JAK/STAT axis^156^. Tumor cells can recruit macrophages into TME by triggering the CCL5/CCR5 signaling pathway and raising the quantity of M2-like macrophages. It was found that CCR5 antagonists can disrupt TAM recruitment via the CCL5/CCR5 pathway, enhancing the clinical outcomes for various cancers, including gastric and pancreatic, by curbing tumor progression and extending patient survival^157–159^. Studies indicate that chemokine antagonists, such as CCR5 antagonists, in combination with conventional chemotherapeutic agents, can help overcome resistance in tumor therapy. A PICCASSO Phase I trial investigating the combination of Pembrolizumab and Maraviroc in metastatic colorectal cancer is currently underway. It has been found feasible and has demonstrated a favorable safety profile.

Furthermore, by targeting M2-like macrophage pro-apoptosis, the number of M2-like macrophages in TME can be directly decreased, thus attenuating tumor drug resistance. For instance, the Chang Wei Qing decoction^160^ has been shown to enhance the apoptotic activity of M2-like TAMs and diminish the production of VEGF and TGF-β, consequently reducing tumor cell tolerance to oxaliplatin.

### Antitumor nanodrugs targeting M2-like TAMs

Nanomaterials have significant potential for enhancing the effectiveness of tumor immunotherapy with the advancement of nanobiotechnology due to their benefits in specific targeted drug transport, accurate localization of drug release, ease of surface functionalization, and high bioavailability of pharmaceuticals^161–163^.

#### Antitumor nanodrugs utilizing TAMs as a delivery medium

TAMs exhibit significant homing properties, able to migrate directionally and accumulate in tumor tissue through the detection of specific signals in the TME. Their integral role in promoting tumor progression, invasion, and metastasis is well-established. Moreover, TAMs also have the capacity to function as vehicles for the delivery of antitumor therapeutics, including drugs, liposomes, and nanoparticles, directly to tumor sites and even into the tumor cells themselves. The homing and delivery capabilities of TAMs determine their potential use as cellular vectors for therapeutic delivery. Leveraging TAMs for the targeted delivery of antitumor nanomedicines is a prospective avenue within the evolving landscape of antitumor therapy.

This TAM-mediated drug delivery strategy is also known as the “Trojan Horse” approach^164^. This technique entails ex vivo loading of nanodrugs into TAMs, which are subsequently reintroduced into the patient. These TAMs migrate to inflammatory sites and are then recruited into the tumor tissue to release the loaded nanomedicines. This strategy can be applied to thermal ablation and radiotherapy of tumors. For instance, Au nanoshells are nanoparticles with a silica core and a thin Au shell. After co-culturing with TAMs, Au nanoshells are phagocytized, internalized within TAMs, and these TAMs carry and accumulate them in the hypoxic core regions of tumors, improving the targeting of gold nanoparticles and enhancing the efficacy of tumor thermal ablation therapy^165^.

Recent years have seen a downtrend in the exploration of M2-like macrophages as vectors for nanomedicine delivery. This paradigm shift can be attributed to M2-like TAMs’ immunosuppressive role in the TME and the difficulties associated with selective targeting. Such constraints not only compound the sophistication of therapeutic conveyance but also risk augmenting tumor advancement. Despite these challenges, the advancement of new research methods and technologies still holds the potential for breakthroughs in future treatment strategies.

#### Antitumor nanodrugs acting on M2-like TAMs

Pro-inflammatory macrophages, known as M1-like TAMs, possess potent phagocytic capabilities and the ability to directly kill tumor cells. Under the influence of relevant chemokines, pro-inflammatory macrophages can efficiently migrate to tumor lesions to exert these functions. Therefore, nanomaterials that target M2-like macrophages to reshape the TAMs phenotype, induce M2-like macrophages to polarize toward M1-like macrophages, and inhibit tumor growth also hold significant research value^166^. Currently, several nanotechnological strategies for acting on M2-like macrophages have been established, including termination of M2-like macrophages recruitment, specific targeting and eliminating M2-like macrophages, and converting M2-like macrophages into M1-like macrophages^167–169^. Recent advances in explicitly enhancing antitumor immune responses by targeting M2-like macrophages with nanomaterials have demonstrated considerable promise.

Chang et al.^170^ designed a tumor-targeting nanoparticle drug carrier, NanoMnSor, which effectively co-delivered oxygen-producing manganese dioxide (MnO2) and sorafenib to HCC. The MnO2 component catalyzed the decomposition of hydrogen peroxide (H2O2) to oxygen, thereby mitigating hypoxic conditions within the TME. Subsequently, NanoMnSor enhanced the presence of CD8+ cytotoxic T cells by polarizing M2-like macrophages into immunologically stimulating M1-like macrophages within tumors, which contributes to the reversal of sorafenib resistance. Li et al.^171^ demonstrated that nanoliposome C6-Ceramide (LipC6) could deplete TAMs and downregulate TAM-regulated ROS signaling pathways. This modulation enhanced CD8+ T cells activity and promoted the release of pro-inflammatory M1 cytokines, including IL-12, IFN-γ, and TNF-α, thereby inducing the activation of M1-like macrophages. Concurrently, LipC6 inhibited the secretion of M2-associated cytokines IL-4, Fizz, and Ym1, which in turn suppressed the activation of M2-like macrophages, thereby diminishing immune tolerance within the hepatocellular carcinoma microenvironment. Scientists are increasingly using structurally modified albumin as a vehicle for drug delivery systems, aimed at selectively activating TLR pathways to target and alter the phenotype of TAMs. TLR7/8 has been identified as a promising target for enhancing the immune response. A TLR7/8 agonist called resiquimod (R848) has been proven to induce the polarization of M2-like to M1-like macrophages. Researchers have developed R848-loaded poly (lactic-co-glycolic acid) (PLGA) nanoparticles, which are subsequently electrostatically adsorbed onto the surface of Escherichia coli strain MG1655. The system promotes tumor cell death by conjugation with DOX, and increases the quantity of T cells within the tumor milieu, thus potentiating antitumor therapeutic efficacy^172^. Beyond their capacity for delivering tiny molecules with precision, nanodrugs can also mediate photodynamics to act directly on M2-like macrophages. A TAM-targeting probe (IRD-αCD206) was created by Zhang et al.^173^ through the conjugation of a CD206 antibody with a near-infrared phthalocyanine dye. This complex exhibited significant efficacy in inhibiting both the proliferation and metastasis of sorafenib-resistant 4T1 breast cancer cells.

### Repolarize M2-like to M1-like macrophages

TAMs exhibit remarkable plasticity, capable of phenotype switching under various factors influences. Therefore, the targeted reprogramming of TAMs from M2 to M1 phenotype represents a critical area of oncological research. To attenuate tumor drug resistance, researchers have undertaken numerous experiments and created a variety of related drugs that induce the polarization from M2-like to M1-like macrophages^174^, including but not limited to CSF-1R antagonists, PI3Kγ inhibitors, bromodomain-containing protein 4 (BRD4) inhibitors, Signal-regulatory protein alpha (SIRPα) inhibitors and histone deacetylase (HDAC) inhibitors.

Pexidartinib is a polygenic tyrosine kinase inhibitor that targets CSF-1R to significantly mitigate macrophage tumor infiltration^175^. Pexidartinib was shown by Omstead^176^ to upregulate BCL-2-associated X protein (BAX), CRISPR-associated protein 3 (Cas3), TNF-α, IFN-γ, and IL-6, and downregulate Ki-67, IL-13, IL-10, TGF-β, and Arg-1. Additionally, Pexidartinib enhanced CD3 + CD8 + T cells infiltration in the TME by inhibiting the CSF-1/CSF-1R axis. Consequently, Pexidartinib could attenuate the polarization of M2-like TAMs, potentially overcoming the esophageal adenocarcinoma (EAC) model’s resistance to PD-1/PD-L1 axis blockade. AZD5153, a specific BRD4 inhibitor, reprograms TAMs from M2 to M1 phenotype, which in turn promotes the secretion of pro-inflammatory cytokines. This secretion cascade activates CD8+ cytotoxic T lymphocytes (CTLs), thereby enhancing the responsiveness of high-grade serous ovarian cancer (HGSOC) to anti-PD-L1 therapy^177^. Evorpacept, a CD47-SIRPα inhibitor, has been shown to enhance antitumor immune response in preclinical models by promoting phagocytosis of macrophages, driving the phenotype shift of M2-like to M1-like TAMs, and boosting cytotoxic T cell effector functions. Evorpacept has also shown encouraging initial combination therapy activity in Phase I clinical trial for solid tumors^178^. Preclinical data suggest that the combination of a CD40 agonist and anti-PD-1/anti-PD-L1 inhibitors improves survival in mouse tumor models compared with the use of either alone. The co-administration of a CD40 agonist and anti-PD-1/anti-PD-L1 inhibitors elevates PD-L1 expression in tumor-infiltrating monocytes and TAMs, biases the TAM populations toward the inflammatory M1 phenotype, thereby inhibiting tumor-induced immune resistance^179^. Kaneda et al.^180^ found in preclinical studies that a PI3K-γ inhibitor could induce a shift in TAMs from M2 to M1 phenotype, restored CD8 + T cell activation and cytotoxicity, thereby inhibiting the growth of checkpoint inhibitor-resistant tumor. In a Phase 1 clinical trial, the PI3K-γ inhibitor IPI-549, used in combination with nivolumab, showed signs of well-tolerated and immunomodulatory clinical activity. Recruitment is presently underway for a subsequent Phase 2 clinical trial to further evaluate efficacy and safety^181^. In vitro experiments demonstrated that dasatinib, a Src inhibitor, promoted M2-like macrophages to polarize toward M1-like macrophages in cisplatin-resistant lung cancer cell lines A549R and H460R by modulating the Src/CD155/MIF axis and reducing the expression of the stem cell markers Notch1 and β-catenin. Research has shown that Src inhibitors are effective in treating individuals with cisplatin-resistant lung cancer by specifically targeting M2-like macrophages^182^. Optimized doses of the HDAC inhibitor Tucidinostat significantly polarized M2-like to M1-like macrophages in three mouse tumor models by activating the NF-B signaling pathway and upregulating CCL5. This led to an increased presence of CD8 + T cells in the TME and reduced tumor resistance to anti-PD-L1 antibodies^183^. The absence of SHP-2 in myeloid cells can activate pro-inflammatory TAMs, transforming the TME from immunosuppressive to immune-stimulatory, thereby enhancing the effectiveness of immunotherapy^184^. Jian Gao’s in vivo research^185^ shows that the SHP2 inhibitor SHP099 targets SHP-2, regulating the STING pathway and boosting type I IFNs, thus remodeling the TME and overcoming tumor resistance. Danvatirsen, a therapeutic antisense oligonucleotide (ASO) specific for STAT3, modifies the TME by rebalancing suppressive and pro-inflammatory macrophages. This action amplifies the efficacy of immune checkpoint blockade and overcomes immunotherapy resistance^186^. Yue et al.^187^ found that anlotinib overcomed bortezomib resistance by promoting the polarization of M2-like TAMs to M1-like TAMs, decreasing tumor vascular function, and accelerating apoptosis in myeloma PDX cells.

A wide range of investigations conducted in recent years have revealed that Traditional Chinese Medicine (TCM) is implicated in the regulation of TAM polarization and the reversal of tumor drug resistance. Traditional Chinese medicine Qi Ling (QL) provides potent anti-prostate tumor benefits. Cao et al.^188^ collected the serum of rats that had been administered QL. It was referred to as QL-serum. They discovered that through the IL-6/STAT3 signaling pathway, QL-serum increased the expression of iNOS and TNF-α, and decreased the expression of IL-10 and CCL22 in a co-culture system of TAMs with paclitaxel-resistant prostate cancer cells, namely DU145-TxR and PC-3-TxR. Thus, M2-like macrophage polarization toward M1-like macrophages was induced, and the resistance of human prostate cancer cells to paclitaxel was diminished. Xu Li’s experiential prescription (XLEP) has been applied in the treatment of NSCLC, which is mainly composed of *Radix adenophorae*, *Radix Glehniae*, *Radix Asparagi*, *Radix Ophiopogonis*, *Schisandra*, *Privet fruit*, *Astragalus*, *Zedoary*. Xu et al.^189^ discovered that XLEP could promote M2-like macrophage polarization to M1-like macrophages and increase the M1-like /M2-like macrophage ratio in EGFR-positive NSCLC cells. This effect was mediated by the downregulation of mTOR, IL-10, TGF-β, and CCL22 and the upregulation of IL-6, CCL2, CCL3, and TNF-α, thereby delaying resistance to the EGFR-TKI inhibitor gefitinib. *Hedyotis diffusa* Willd (HDW) targeted and modulated the TGF-β signaling pathway in CRC cells to overcome chemoresistance. However, additional experimental studies are still required to establish whether HDW regulates M2-like macrophage polarization^190^. Zhi Zhen Formula was able to prevent CRC cells from developing resistant to 5-FU through reducing p-STAT3 production, a critical protein in the STAT3 pathway within TAMs. This effect was accomplished by inhibiting TAM polarization towards the M2 phenotype. and by suppressing the activation of the Hedgehog signaling pathway in CRC cells^191^. Tripterygium lactone (TP), a targeted inhibitor of the PI3K/Akt/NF-κB pathway, decreased the expression of MMP-2, MMP-9, and VEGF, which stimulated TAM polarization from the M2 to M1 phenotype. This shift increased the M1/M2 ratio and reduced the resistance of OCA cells to cisplatin^192^.

## Discussion

The use of drug therapies, including chemotherapy, targeted therapy, and immunotherapy, is crucial for tumor treatment. However, in clinical practice, a majority of patients will develop drug resistance at the later stage of treatment, leading to the failure of antitumor therapy^193–195^. Reducing tumor resistance and enhancing anticancer treatment efficacy remain significant challenges. Recent advances in the study of the TME and TAMs have elucidated that M2-like TAMs contribute to an immunosuppressive TME, subsequently promoting drug resistance. M2-like TAMs can promote tumor resistance through several mechanisms such as regulating cytokines, signaling pathways, exosomes, the immune microenvironment, angiogenesis, and tumor stem cells. Based on these related mechanisms, it’s feasible to formulate drugs targeting M2-like TAMs specifically. Combining these drugs with chemotherapy, targeted therapy, immunotherapy, and other antitumor medications in clinical treatment holds promise for overcoming tumor drug resistance and enhancing prognosis. It will take further clinical trials to validate the potential of targeting M2-like TAMs in combination with different therapies.

### Reporting summary

Further information on research design is available in the Nature Research Reporting Summary linked to this article.

### Supplementary information

Reporting summary
